# Supplementary material for: Lithium diffusion-controlled Li-Al alloy negative electrode for all-solid-state battery
Source: Nat Commun. 2025 Oct 31;16:9629. doi: 10.1038/s41467-025-64386-y (PMC12579251; doi:10.1038/s41467-025-64386-y)
Supplement: Supplementary file 1 — Supplementary information [file 41467_2025_64386_MOESM1_ESM.pdf]

Supplementary Information

Lithium Diffusion-Controlled Li-Al Alloy Negative Electrode for All-Solid-State Battery

Yuju Jeon,[1] Dong Ju Lee,[1] Hongkui Zheng,[2] Sesha Sai Behara,[3] Jung-Pil Lee,[4] Junlin Wu,[5] Feng Li,[1] Wei Tang,[1] Lanshuang Zhang,[5] Yu-Ting Chen,[5] Dapeng Xu,[1] Jiyoung Kim,[4] Min-Sang Song,[4] Anton Van der Ven,[3]* Kai He[2]* and Zheng Chen[1,5]*

[1]Aiiso Yufeng Li Family Department of Chemical and Nano Engineering, University of California, San Diego, 9500 Gilman Drive, La Jolla, CA 92093, USA

[2]Department of Materials Science and Engineering, University of California, Irvine, CA 92697, USA

[3]Materials Department, University of California, Santa Barbara, Santa Barbara, CA 93106, United States

[4]LG Energy Solution, Ltd., LG Science Park, Magokjungang 10-ro, Gangseo-gu, Seoul 07796, Republic of South Korea

[5]Program of Materials Science and Engineering, University of California, San Diego, 9500 Gilman Drive, La Jolla, CA 92093, USA

* Corresponding author: zhc199@ucsd.edu, kai.he@uci.edu, avdv@ucsb.edu

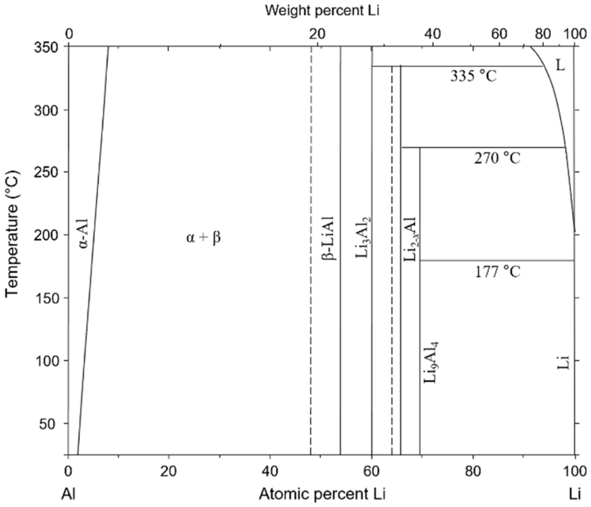

Supplementary Figure 1. Binary Li-Al phase diagram.

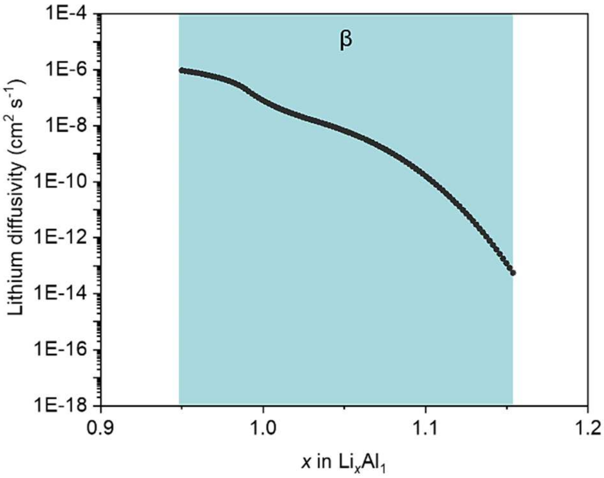

Supplementary Figure 2. Li diffusivity calculation at β phase.

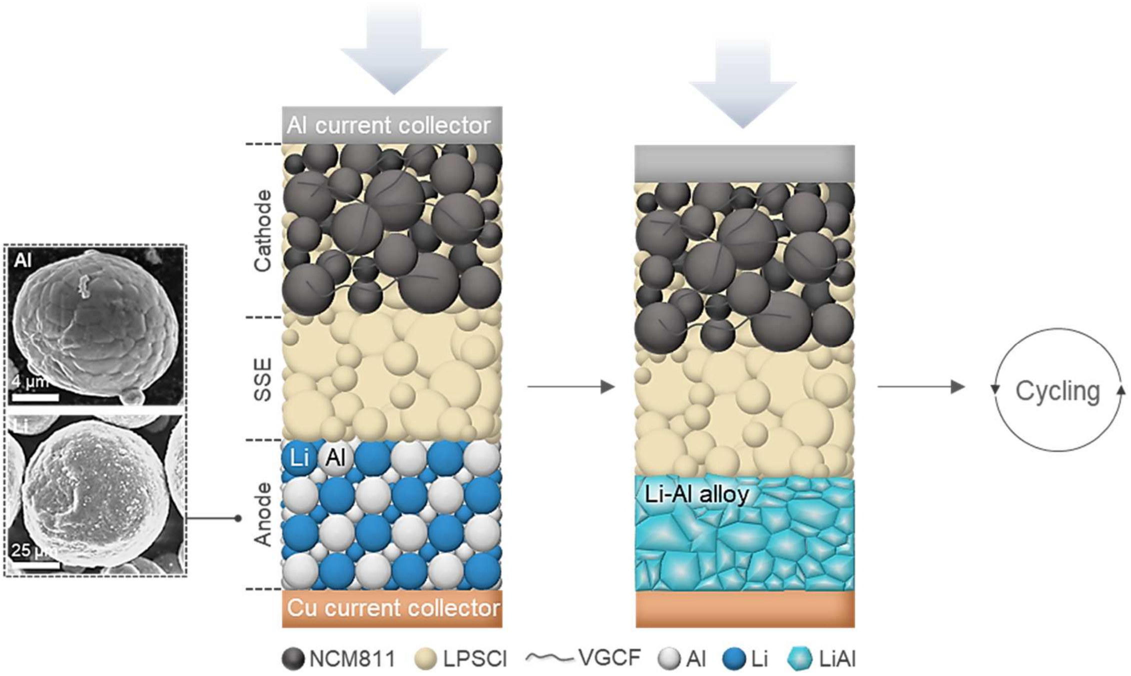

Supplementary Figure 3. In situ prelithiation of pure Al with Li powder in cell assembly procedure.

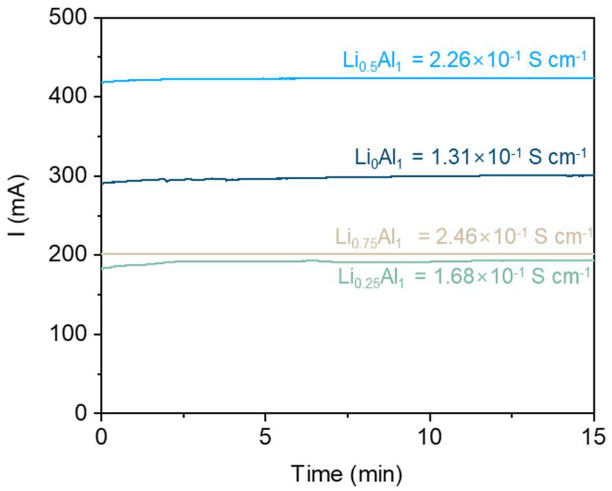

Supplementary Figure 4. Electronic conductivity measurement of Al, $Li_{0.25}Al_1$, $Li_{0.5}Al_1$ and $Li_{0.75}Al_1$ negative electrodes. Direct current (DC) polarization curve was observed at an applied voltage of 50 mV and stack pressure of 75 MPa. Testing temperature: 25 ± 1 °C.

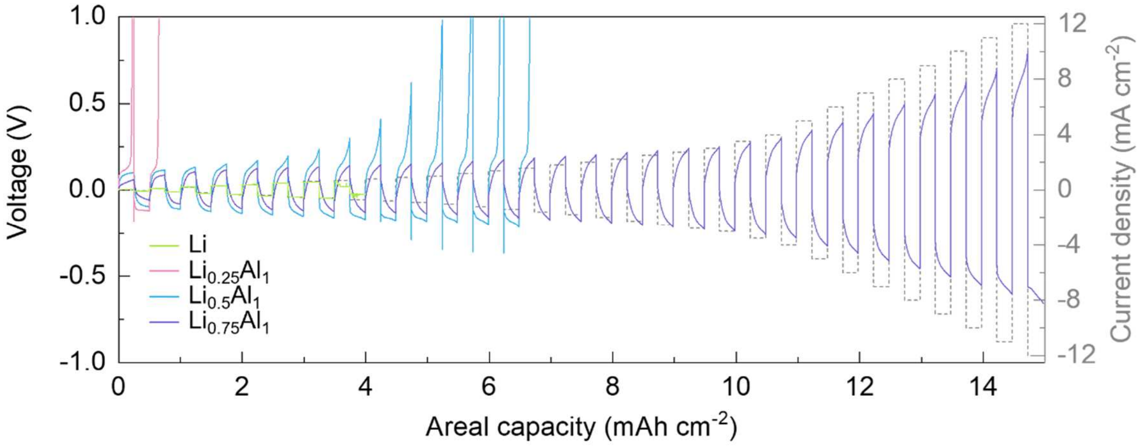

Supplementary Figure 5. Critical-current-density test of symmetric cells with Li, $Li_{0.25}Al_1$, $Li_{0.5}Al_1$ and $Li_{0.75}Al_1$ electrodes. Lithiation and delithiation were conducted up to 0.25 mAh cm^{-2} at each current density. Stack pressure: 10 MPa for Li and 75 MPa for Li-Al alloy electrodes. Testing temperature: 25 ± 1 °C.

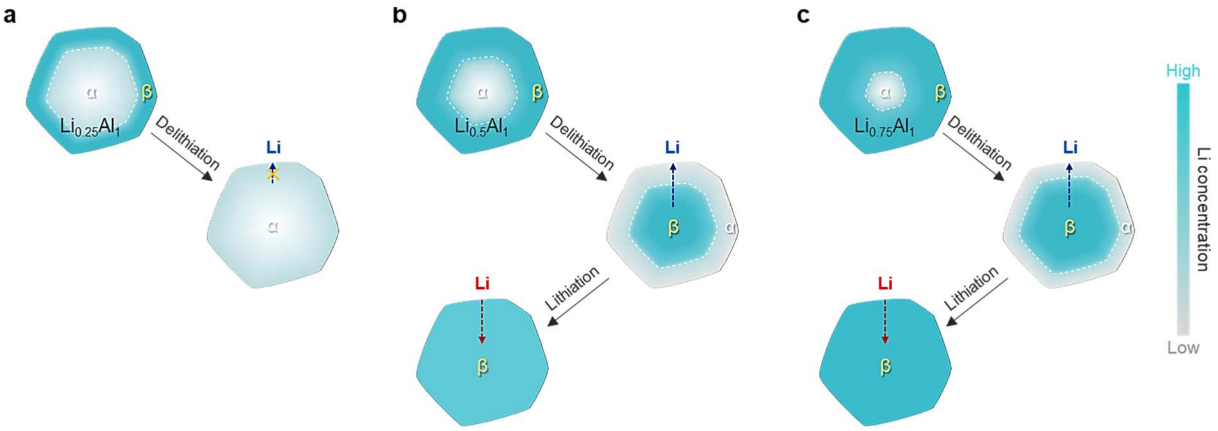

Supplementary Figure 6. Schematics of the phase development during cycling of symmetric cells with Li-Al alloy electrodes. a $Li_{0.25}Al_1$. **b** $Li_{0.5}Al_1$. **c** $Li_{0.75}Al_1$.

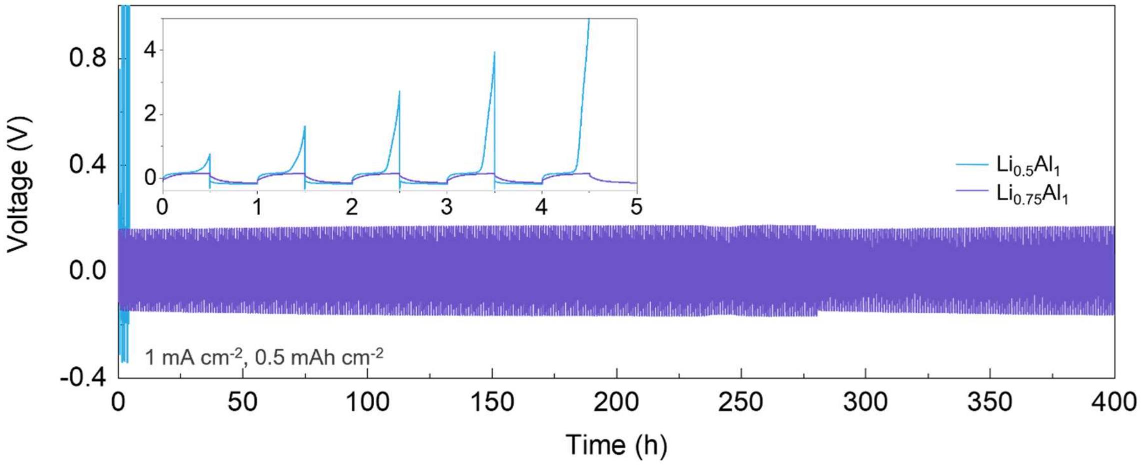

Supplementary Figure 7. Long-term cyclability of symmetric cells with $Li_{0.5}Al_1$ and $Li_{0.75}Al_1$ negative electrodes. The inset shows magnified voltage profiles of the $Li_{0.5}Al_1$ symmetric cell during the first 5 hours. Lithiation and delithiation were conducted at 1 mA cm^{-2} up to 0.5 mAh cm^{-2}. Stack pressure: 75 MPa. Testing temperature: 25 ± 1 °C.

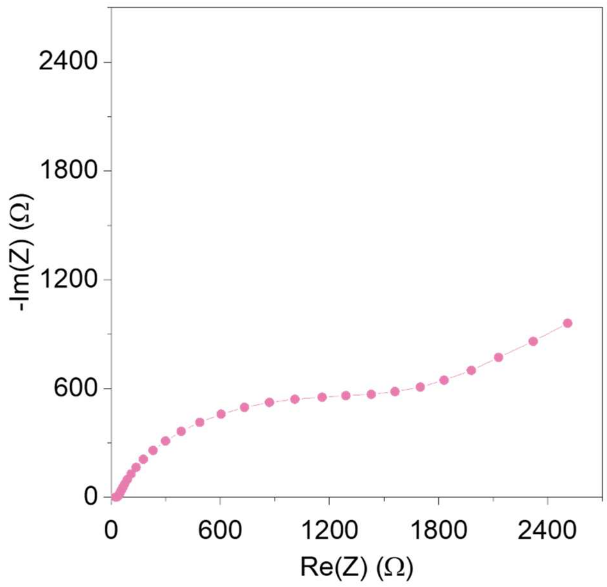

Supplementary Figure 8. Electrochemical impedance spectra of 200 h-cycled $Li_{0.25}Al_1$ alloy negative electrode at stack pressure of 75 MPa. Testing temperature: 25 ± 1 °C.

a

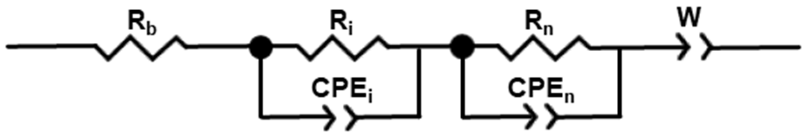

b

	R_{bulk} (R_b, Ω)	$R_{interface}$ (R_i, Ω)	$R_{negative\ electrode}$ (R_n, Ω)	χ^2
Pristine $Li_{0.75}Al_1$	34.7	2.1	12.6	0.0011
Cycled $Li_{0.75}Al_1$	23.9	0.7	17.5	0.0005
Pristine $Li_{0.5}Al_1$	35.9	2.3	12.6	0.0030
Cycled $Li_{0.5}Al_1$	24.6	4.6	27.5	0.0006
Pristine $Li_{0.25}Al_1$	26.8	6.6	29.4	0.0006
Cycled $Li_{0.25}Al_1$	25.1	4.8	1450	0.0052

Supplementary Figure 9. a Equivalent circuit used for fitting EIS spectra in Fig. 3b. **b** Fitting results.

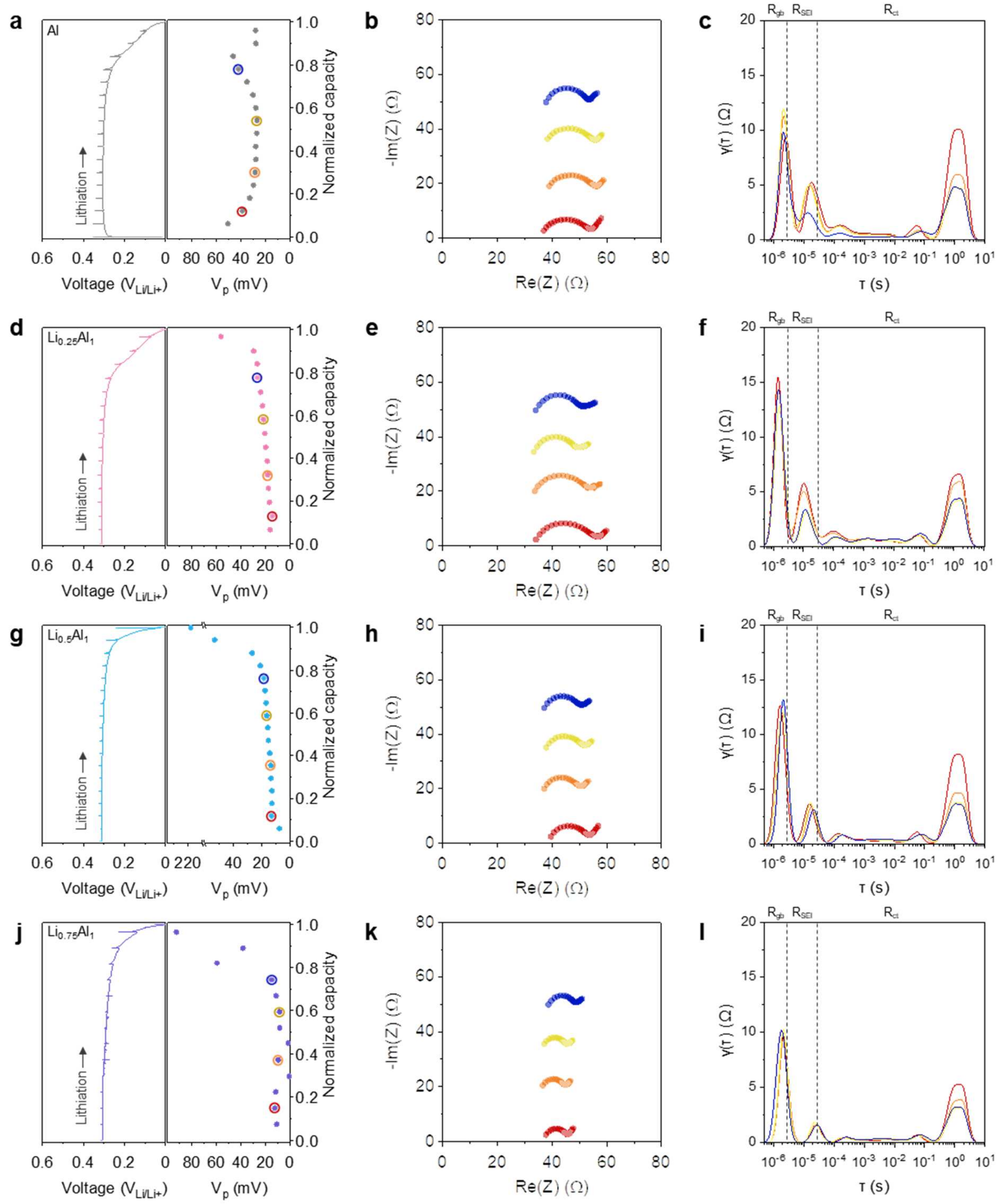

Supplementary Figure 10. Analysis of GITT, in situ EIS, and DRT on lithiation of pure Al and Li-Al alloy negative electrodes in Li metal half cell. a - c Pure Al. **d - f** $Li_{0.25}Al_1$. **g - i** $Li_{0.5}Al_1$. **j - l** $Li_{0.75}Al_1$. GITT was conducted with galvanostatic titration at 0.1 mA cm^{-2} for 2 hours, followed by relaxation at open circuit for 1 hour. EIS was measured immediately after each titration within a frequency range from 5 MHz to 0.5 MHz. The EIS corresponding to the red, orange, yellow and green circles were used for b, c, e, f, h, I, k and l, excluding the measurements in the unstable range of the normalized capacity below 0.1 and above 0.9. Stack pressure: 50 MPa. Testing temperature: 25 ± 1 °C.

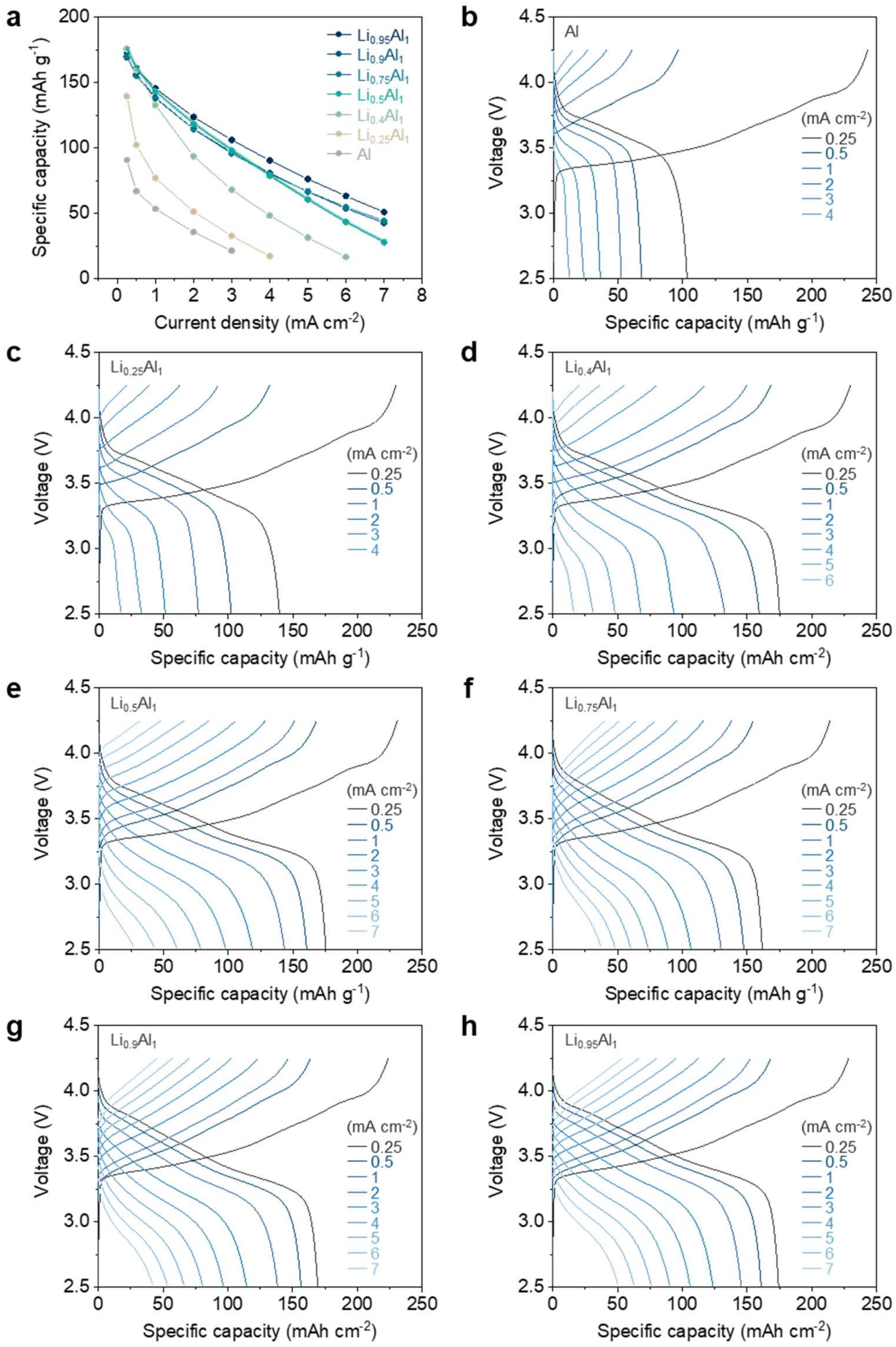

Supplementary Figure 11. Rate capability test for the kinetics improvement of Li_xAl_1 alloy electrodes along with prelithiation degree increase in NCM811-based full cells. a Specific capacity comparison among Li_xAl_1 alloy electrodes at each current density. **b** Pure Al. **c** $Li_{0.25}Al_1$. **d** $Li_{0.4}Al_1$. **e** $Li_{0.5}Al_1$. **f** $Li_{0.75}Al_1$. **g** $Li_{0.9}Al_1$. **h** $Li_{0.95}Al_1$. Stack pressure: 75 MPa. N/P ratio: 2. Testing temperature: 25 ± 1 °C.

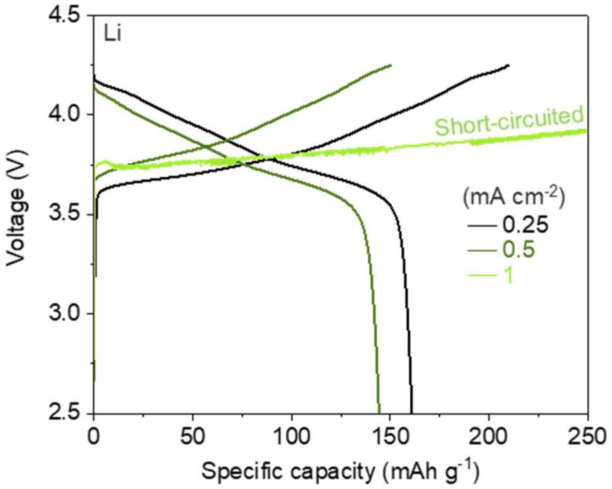

Supplementary Figure 12. Rate capability of Li||NCM811 full cell. Stack pressure: 10 MPa. Testing temperature: 25 ± 1 °C.

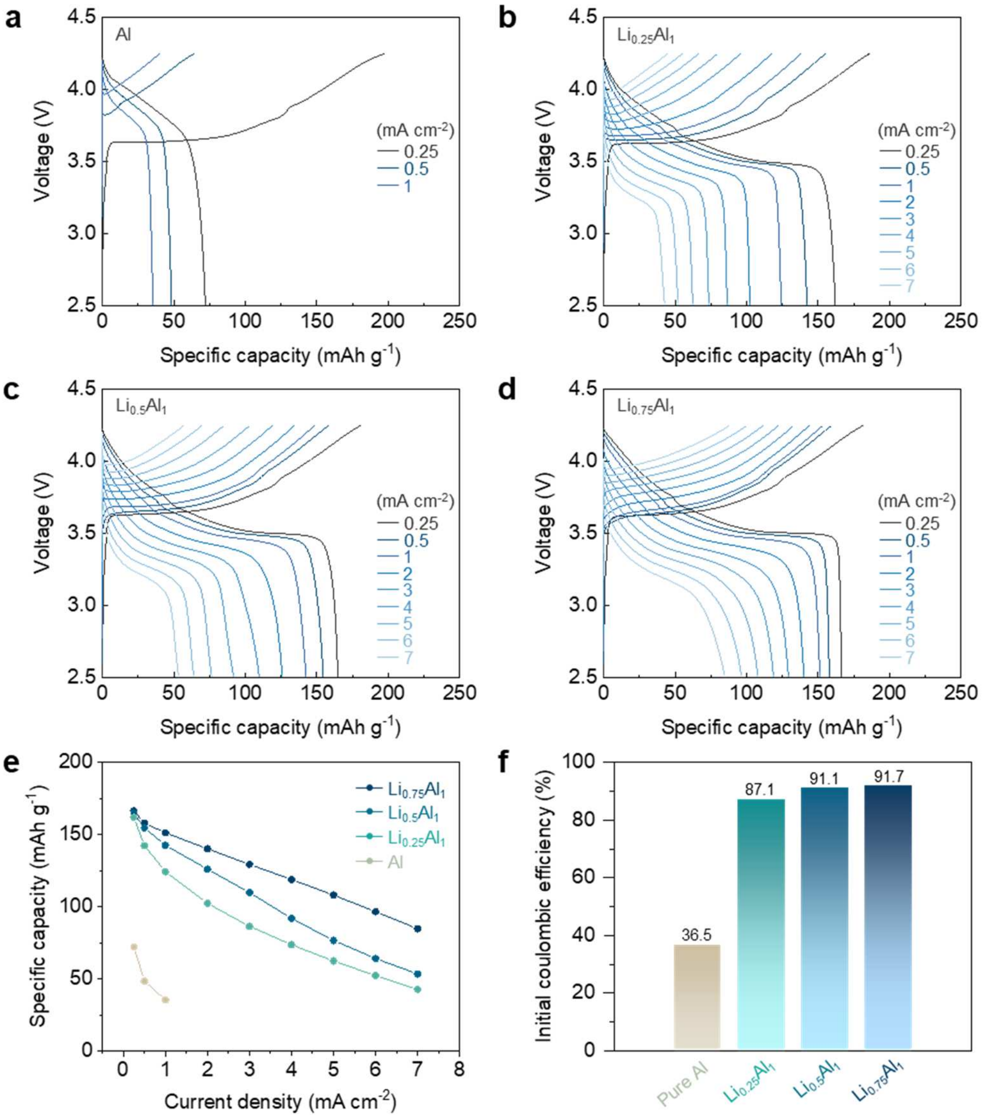

Supplementary Figure 13. Rate capability test for the kinetics improvement of Li_xAl_1 alloy electrodes in LCO-based full cells. a-d Voltage profiles **a** Pure Al. **b** $Li_{0.25}Al_1$. **c** $Li_{0.5}Al_1$. **d** $Li_{0.75}Al_1$. **e** Specific capacity comparison at each current density. **f** Initial coulombic efficiency. Stack pressure: 75 MPa. N/P ratio: 2. Testing temperature: 25 ± 1 °C.

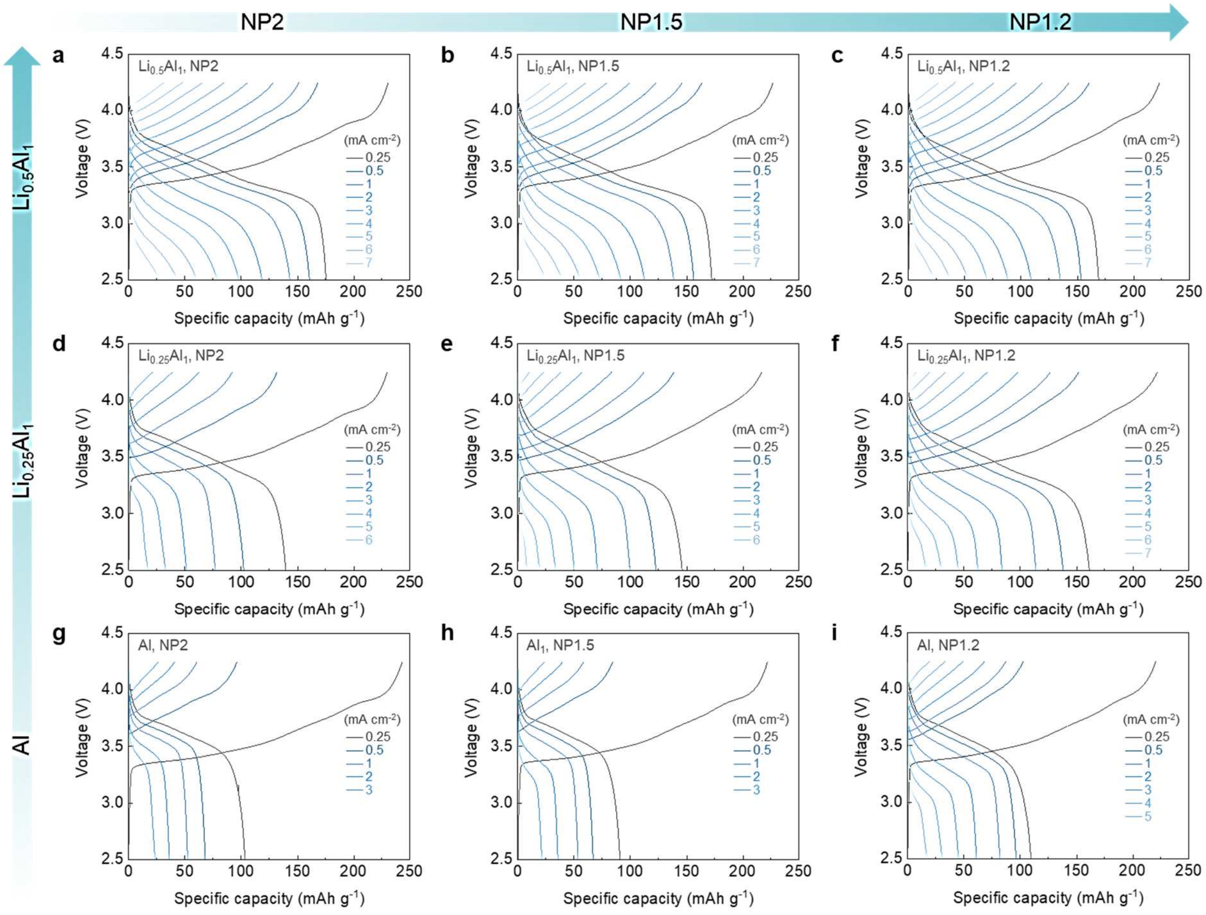

Supplementary Figure 14. Rate capability test for the kinetics improvement along with prelithiation degree and NP ratio. a-c $Li_{0.5}Al_1$ alloy negative electrode at different NP ratios. **a** NP2. **b** NP1.5. **c** NP1.2. **d-f** $Li_{0.25}Al_1$ alloy negative electrode at different NP ratios. **d** NP2. **e** NP1.5. **f** NP1.2. **g-i** Pure Al negative electrode at different NP ratios. **g** NP2. **h** NP1.5. **i** NP1.2. Stack pressure: 75 MPa. Testing temperature: 25 ± 1 °C.

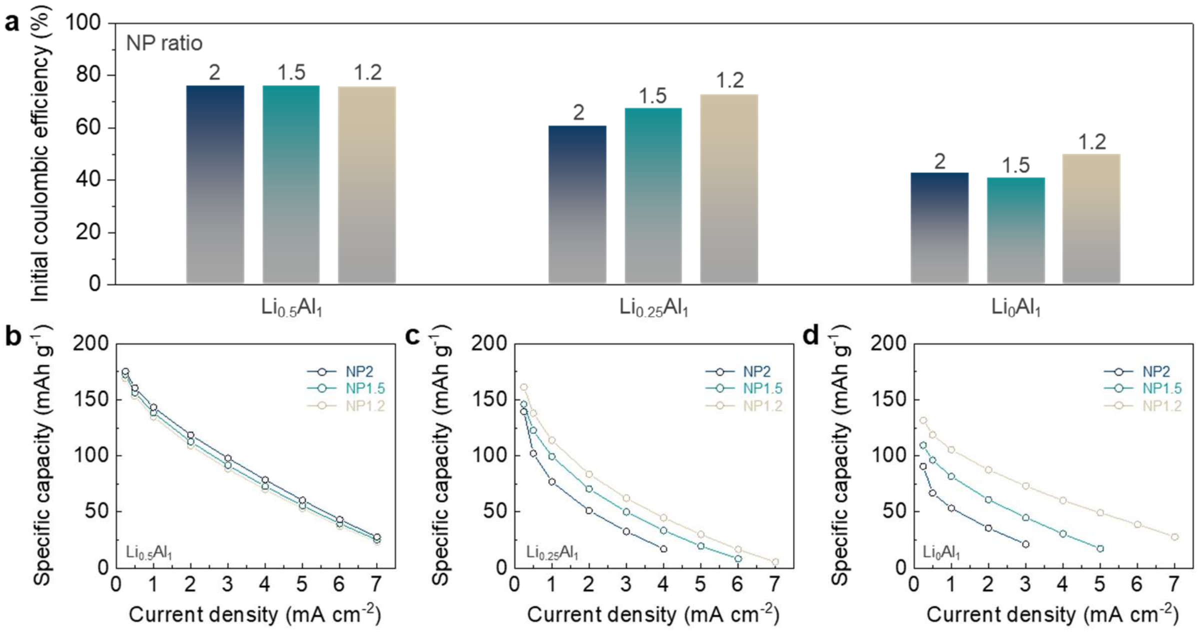

Supplementary Figure 15. Comparison of initial coulombic efficiency and specific capacities along with prelithiation degree and NP ratio. a Initial coulombic efficiency comparison among $Li_{0.5}Al_1$, $Li_{0.25}Al_1$ and pure Al negative electrodes at NP ratio of 2, 1.5 and 1.2. **b-d** Specific capacity comparison among NP ratios of 2, 1.5 and 1.2. **b** $Li_{0.5}Al_1$. **c** $Li_{0.25}Al_1$. **d** Pure Al. Stack pressure: 75 MPa. Testing temperature: 25 ± 1 °C.

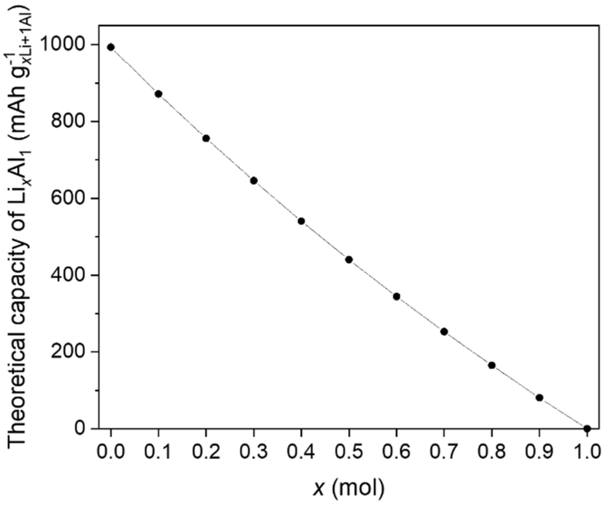

Supplementary Figure 16. Theoretical specific capacity of Li-Al alloy negative electrode according to Li concentration, x.

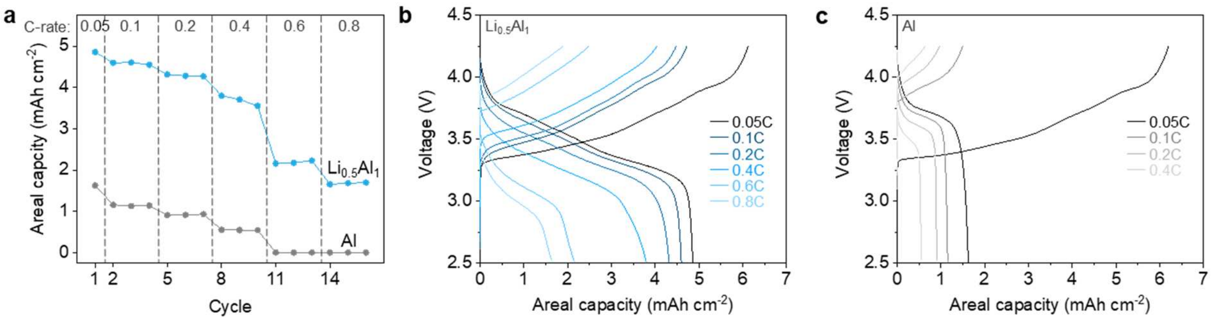

Supplementary Figure 17. Rate capability of $Li_{0.5}Al_1$ and pure Al with high-loading NCM811 positive electrode (1C = 5 mA cm^{-2}). a Areal capacities at various C-rates. **b and c** Voltage profiles at each C-rate. **b** $Li_{0.5}Al_1$. **c** Pure Al. Stack pressure: 75 MPa. N/P ratio: 2. Testing temperature: 25 ± 1 °C.

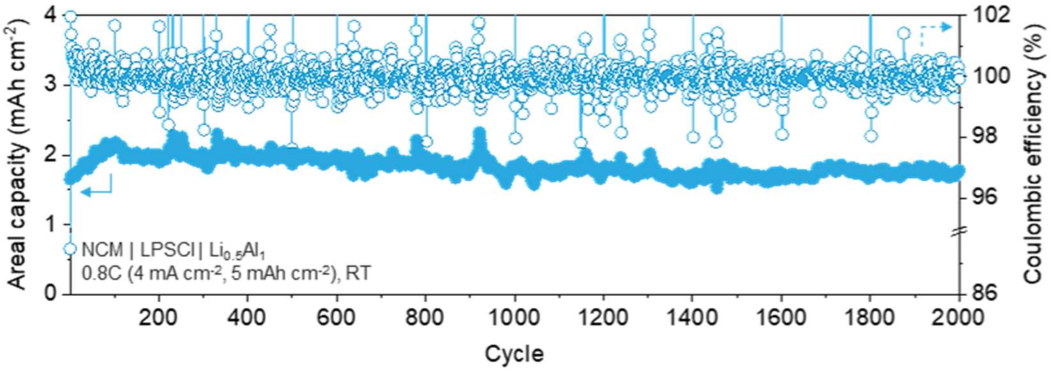

Supplementary Figure 18. Long-term cyclability at 0.8C (1C = 5 mA cm^{-2}). All the data points were included. Stack pressure: 75 MPa. N/P ratio: 2. Testing temperature: 25 ± 1 °C.

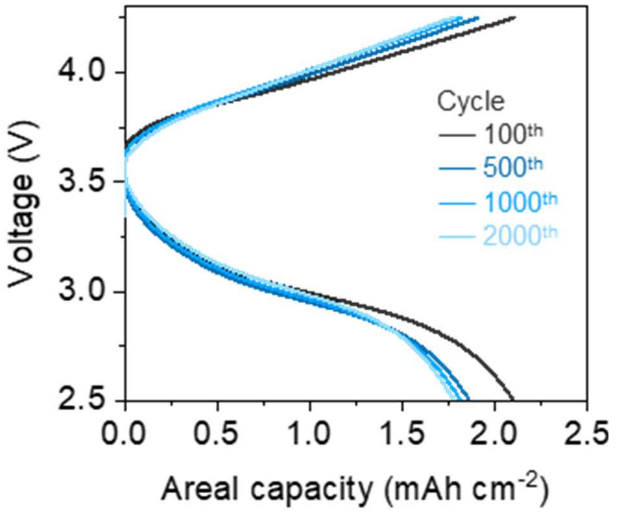

Supplementary Figure 19. Voltage profiles at 100th, 500th, 1000th and 2000th cycle of long-term cyclability at 0.33C in Fig. 4c. Stack pressure: 75 MPa. N/P ratio: 2. Testing temperature: 25 ± 1 °C.

a

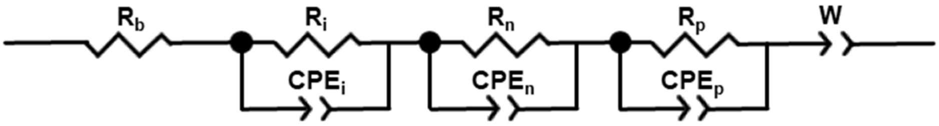

b

Cycle	R_{bulk} (R_b, Ω)	$R_{interface}$ (R_i, Ω)	$R_{negative electrode}$ (R_n, Ω)	$R_{positive electrode}$ (R_p, Ω)	χ^2
100th	25.4	4.6	37.3	67.1	0.00054
500th	25.9	6.1	37.3	68.4	0.00077

Supplementary Figure 20. EIS spectra fitting. a Equivalent circuit used for fitting EIS spectra in Figure 4d. **b** Fitting results.

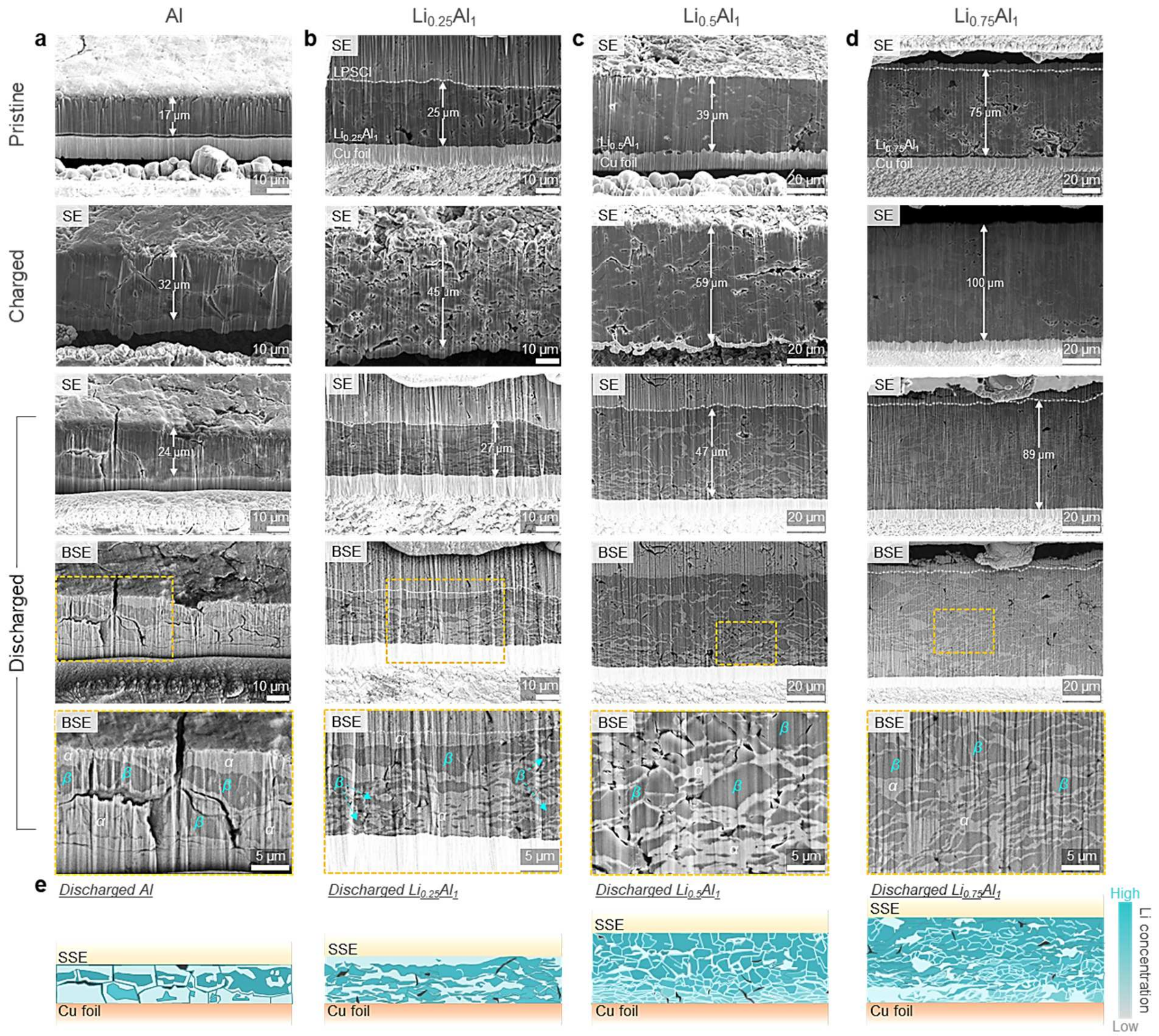

Supplementary Figure 21. Cross-sections of pure Al and Li-Al alloy negative electrodes in the pristine, 1st charged, and 1st discharged states. **a** Pure Al. **b** $Li_{0.25}Al_1$. **c** $Li_{0.5}Al_1$. **d** $Li_{0.75}Al_1$. SE and BSE indicate secondary electron mode and backscattered electron mode, respectively. The area of yellow box was enlarged to high-magnification images at the rightmost row. **e** Schematics of the corresponding discharged Li-Al alloy negative electrodes.

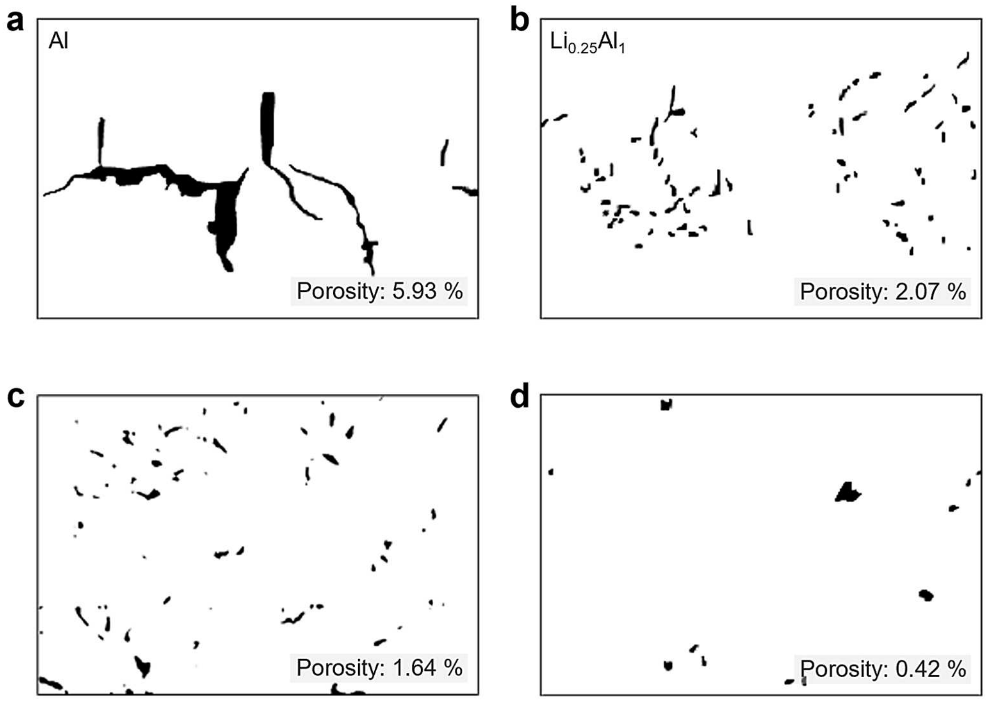

Supplementary Figure 22. Porosity of pure Al and Li-Al alloy negative electrodes in the 1st discharged state. **a** Pure Al. **b** $Li_{0.25}Al_1$. **c** $Li_{0.5}Al_1$. **d** $Li_{0.75}Al_1$.

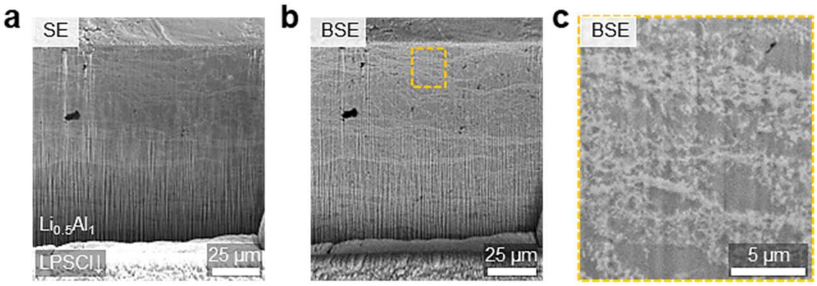

Supplementary Figure 23. Cross-sections of discharged $Li_{0.5}Al_1$ after 2000 cycles. **a** Secondary electron mode. **b** Backscattered electron mode. **c** High-magnification image of the yellow region boxed in b.

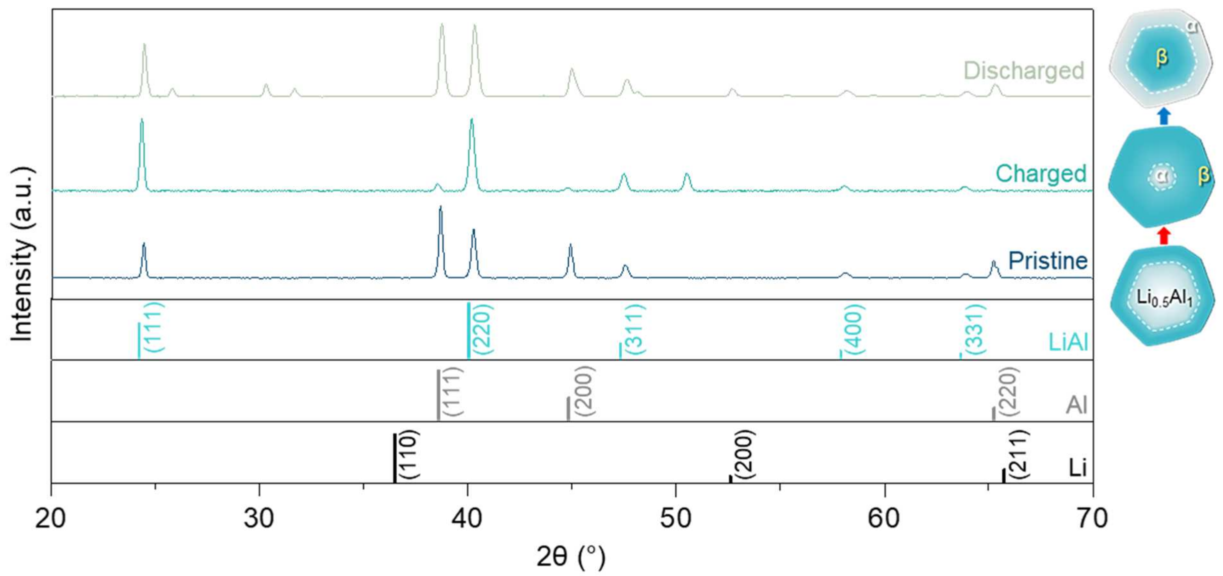

Supplementary Figure 24. XRD pattern of $Li_{0.5}Al_1$ negative electrode in pristine, lithiated and delithiated states. *: LPSCl residue on the surface of the delithiated $Li_{0.5}Al_1$ negative electrode.

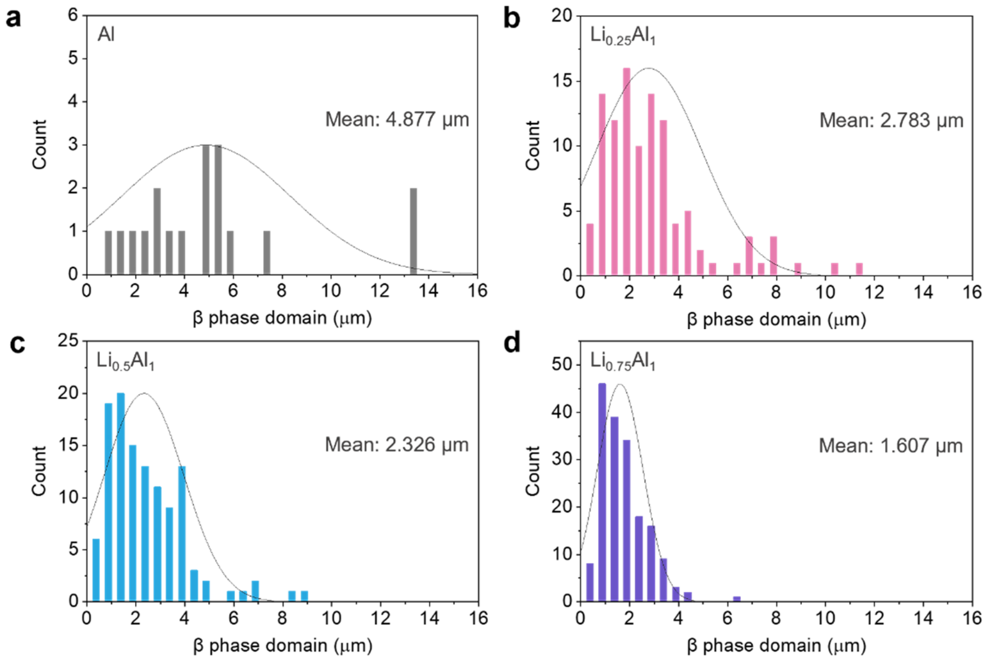

Supplementary Figure 25. Size distribution of β-phase domains within the 1st discharged state. **a** Pure Al. **b** $Li_{0.25}Al_1$. **c** $Li_{0.5}Al_1$. **d** $Li_{0.75}Al_1$.

Supplementary Table 1. Electrochemical, volume change, diffusion coefficient, hardness, abundance of elements and cost properties of various alloy materials.

	**Specific capacity (mAh g^{-1})**	**Lithiated phase**	**Li diffusion coefficient (cm^2 s^{-1})**	**Volume change (%)**	**Vickers hardness [f] (HV)**	**Elemental abundance in the earth's crust (%)**	**Cost (\$ kg^{-1})**
**Al**	**993[1]**	**LiAl[1]**	**10^{-7} – 10^{-9} [2-4, a]**	**96[5]**	**35[6]**	**8.1[7]**	**2.6[g]**
Si	3579[1]	$Li_{15}Si_4$[1]	10^{-9} – 10^{-12} [8-10]	280[1]	1130[11]	29.5[7]	1.4[h]
Mg	2150[1]	$Li_{1.95}Mg$[1]	~10^{-7} [12, b]	125[1]	21[13]	1.9[7]	2.2[h]
In	1012[14]	$Li_{13}In_3$[14]	~10^{-6} [4, c]	105[15, c]	10[16]	2.5×10^{-5} [7]	349.5[h]
Sn	993[1]	$Li_{4.4}Sn$[1]	~10^{-7} [17, d]	244[1]	7[6]	2.5×10^{-4} [7]	35.1[g]
Ag	670[1]	$Li_{2.7}Ag$[1]	~10^{-8} [18,e]	236[1]	91[16]	0.7×10^{-5} [7]	1098.1[i]

[a] Includes the Li diffusion coefficient calculated in this study

[b] Measured for Li_xMg ($1.34 \leq x \leq 3.65$)

[c] Measured for LiIn

[d] Measured for $Li_{2.33}Sn$

[e] Measured for Li_xAg ($4.73 \leq x \leq 5.18$)

[f] Measured for pure materials

[g] Element price as of March 2025 from London Metal Exchange

[h] Element price as of March 2025 from Shanghai Metals market

[i] Element price as of March 2025 from Bloomberg

Supplementary Table 2. Key metrics used to calculate energy density and specific energy in Figure 3g.

Cell Model Parameters					
		Li_0Al_1	$Li_{0.25}Al_1$	$Li_{0.5}Al_1$	$Li_{0.75}Al_1$
**Positive electrode**	NCM811 capacity (mAh g^{-1})	90.7	139.4	175.4	170
	NCM811 ratio (%)	77			
	Mass loading (mg cm^{-2})	30			
	Porosity (%)	20			
	Thickness (μm)	120			
	Areal capacity (mAh cm^{-2})	2.7	4.2	5.3	5.1
	Al foil thickness (μm)	10			
	Al foil mass (mg cm^{-2})	2.7			
**SSE**	Thickness (μm)	30			
	Mass loading (mg cm^{-2})	4.92			
**Negative electrode**	Capacity (mAh g^{-1})	993.3	700	440.1	208.2
	NP ratio	1.1			
	Active material ratio (%)	99.9			
	Alloy metal mass loading (mg cm^{-2})	6.6	9.4	15	31.7
	Li metal mass loading (mg cm^{-2})		0.6	1.7	5.1
	Porosity (%)	5	5	5	5
	Thickness at charged (μm)	23.3	32.9	55	88
	Areal capacity (mAh cm^{-2})	6.6			
	Cu foil thickness (μm)	10			
	Cu foil mass (mg cm^{-2})	8.96			
**Pouch**	Packaging Thickness (μm)	240			
	Packaging mass (mg cm^{-2})	36.7			
	Model area (cm^2)	25			
	Layers	20			
	Total Thickness (μm)	4105.5	4298.3	4740	5400
	Nominal Voltage (V)	3.4			
	Cell capacity (mAh)	1360.8	2091.5	2631.2	2550.8
	Energy density (Wh L^{-1})	450.8	661.7	754.9	642.4
	Specific energy (Wh kg^{-1})	168.1	245.9	282.2	216.5

References

1. Obrovac, M. N., Chevrier, V. L. Alloy negative electrodes for Li-ion batteries. *Chem. Rev.* **114**, 11444-11502 (2014).

2. Jow, T. R., Liang, C. C. Lithium-aluminum electrodes at ambient temperatures. *J. Electrochem. Soc.* **129**, 1429 (1982).

3. Armstrong, R. D., Brown, O. R., Ram, R. P., Tuck, C. D. Lithium electrodes based upon aluminium and alloy substrates I. Impedance measurements on aluminium. *J. Power Sources* **28**, 259-267 (1989).

4. Tarczon, J. C., Halperin, W. P., Chen, S. C., Brittain, J.O. Vacancy antistructure defect interaction diffusion in beta-LiAl and beta-LiIn. *Mat. Sci. Eng. A-Struct.* **101**, 99-108 (1988).

5. Liu, Y., *et al.* Aluminum foil negative electrodes with multiphase microstructure for all-solid-state Li-ion batteries. *Nat. Commun.* **14**, 3975 (2023).

6. Li, H., *et al.* Circumventing huge volume strain in alloy anodes of lithium batteries. *Nat. Commun.* **11**, 1584 (2020).

7. Yaroshevsky, A. A. Abundances of chemical elements in the Earth's crust. *Geochem. Int.* **44**, 48-55 (2006).

8. Ding, N., *et al.* Determination of the diffusion coefficient of lithium ions in nano-Si. *Solid State Ion.* **180**, 222-225 (2009).

9. Pharr, M., Zhao, K., Wang, X., Suo, Z., Vlassak, J. J. Kinetics of initial lithiation of crystalline silicon electrodes of lithium-ion batteries. *Nano Lett.* **12**, 5039-5047 (2012).

10. Tan, D. H. S., *et al.* Carbon-free high-loading silicon anodes enabled by sulfide solid electrolytes. *Science* **373**, 1494-1499 (2021).

11. Walls, M. G., Chaudhri, M. M., Tang, T. B. STM profilometry of low-load Vickers indentations in a silicon crystal. *J. Phys. D Appl. Phys.* **25**, 500-507 (1992).

12. Shi, Z., Liu, M., Naik, D., Gole, J. L. Electrochemical properties of Li–Mg alloy electrodes for lithium batteries. *J. Power Sources* **92**, 70-80 (2001).

13. Lu, Y., *et al.* Effects of minor gadolinium addition and T4 heat treatment on microstructure and properties of magnesium. *Adv. Eng. Mater.* **24**, 2200966 (2022).

14. Songster, J., Pelton, A. The In-Li (indium-lithium) system. *J. Phase Equilib.* **12**, 37-41 (1991).

15. Zhang, W., *et al.* (Electro)chemical expansion during cycling: monitoring the pressure changes in operating solid-state lithium batteries. *J. Mater. Chem. A* **5**, 9929-9936 (2017).

16. Negm, S. E., Mady, H., Bahgat, A. A. Influence of the addition of indium on the mechanical creep of Sn–3.5%Ag alloy. *J. Alloys Compd.* **503**, 65-70 (2010).

17. Wang, J., Raistrick, I. D., Huggins, R.A. Behavior of some binary lithium alloys as negative electrodes in organic solvent-based electrolytes. *J. Electrochem. Soc.* **133**, 457 (1986).

18. Jin, S., *et al.* Solid–solution-based metal alloy phase for highly reversible lithium metal anode. *J. Am. Chem. Soc.* **142**, 8818-8826 (2020).
