# Supplementary material for: Lithium diffusion-controlled Li-Al alloy negative electrode for all-solid-state battery
Source: Nat Commun. 2025 Oct 31;16:9629. doi: 10.1038/s41467-025-64386-y (PMC12579251; doi:10.1038/s41467-025-64386-y)
Supplement: Supplementary file 3 — Description of Additional Supplementary Files [file 41467_2025_64386_MOESM3_ESM.pdf]

Description of Additional Supplementary Files

Supplementary Data 1. NEB calculation parameters, and the initial and final configurations of α-Al.

Supplementary Data 2. NEB calculation parameters, and the initial and final configurations of β-LiAl.
